# Mediating and Moderating Factors of Adherence to Nutrition and Physical Activity Guidelines, Breastfeeding Experience, and Spousal Support on the Relationship between Stress and Quality of Life in Breast Cancer Survivors

**DOI:** 10.3390/ijerph17207532

**Published:** 2020-10-16

**Authors:** Nam Mi Kang, Won-Ho Hahn, Suyeon Park, Jung Eun Lee, Young Bum Yoo, Chung Ja Ryoo

**Affiliations:** 1 Department of Nursing, Research Institute for Biomedical & Health Science, Konkuk University, Chungju 27478, Korea; nmkang03@kku.ac.kr; 2 Department of Pediatrics, School of Medicine, Soon Chun Hyang University, Seoul Hospital, Seoul 04401, Korea; 3 Department of Biostatistics, Soon Chun Hyang Medical Center, Seoul 04401, Korea; suyeon1002@schmc.ac.kr; 4 Department of Food and Nutrition, Seoul National University, Seoul 08826, Korea; jungelee@snu.ac.kr; 5 Department of Surgery, Konkuk University Medical Center, Seoul 05030, Korea; 0117652771@kuh.ac.kr; 6 Graduate School of Nursing, Konkuk University, Chungju 27478, Korea; rcj1109@kku.ac.kr

**Keywords:** American cancer society guidelines on nutrition and physical activity for cancer survivors, breast cancer survivors, breastfeeding, health-related quality of life, spousal support, stress

## Abstract

Spousal support may attenuate stress in breast cancer survivors and improve their health-related quality of life (HRQoL). However, there is limited evidence of a relationship between spousal support, stress, and HRQoL in Asian populations. The current study examined whether spousal support, adherence to the American Cancer Society (ACS) Guidelines on Nutrition and Physical Activity for Cancer Survivors, and breastfeeding experience mediated or moderated the relationship between stress and HRQoL in Korean breast-cancer survivors. Between June 2016 and May 2018, 144 Korean women who survived breast cancer were recruited for the current cross-sectional study. Structured questionnaires and medical records were used to collect data. Structural equation modeling was used to examine mediating and moderating factors. Spousal support buffered the adverse effect of stress on HRQoL (β = −0.22 for stress→spousal support; β = 0.27 for spousal support→physical HRQoL; β = 0.40 for spousal support→mental HRQoL). We found that adherence to ACS guidelines moderated the association between stress and HRQoL (β = −0.14 for stress→mental HRQoL in high ACS adherence; β = −0.79 for stress→mental HRQoL in low ACS adherence). Moreover, beta coefficients were −0.22 for stress→mental HRQoL in women with breastfeeding experience, and −0.71 in those without breastfeeding experience. In conclusion, spousal support mediated the association between stress and HRQoL and this association was moderated by both adherence to ACS guidelines and breastfeeding experience.

## 1. Introduction

Breast cancer currently impacts more than one in ten women globally. In 2018, approximately 25% of cases of breast cancer in women were newly diagnosed, with 2.1 million new cases worldwide [1]. Though breast cancer in women represents one of the top three cancers in terms of incidence, in Korea the breast cancer survival rate has been increasing in recent decades with reports that the five-year survival rate was 79.2% in 1993–1995, and increased steadily to 93.2% in 2013–2017. Moreover, the 10-year survival rate increased from 71.8% in 1993–1995 to 87.7% in 2008–2012 [2]. These remarkable changes suggest that socio-demographic factors such as the supportive strategies provided by family and society enhance the quality of life (QoL) of breast cancer patients [3]. The improvements in survival rate highlight the importance of long-term QoL for breast cancer survivors in parallel with greater supportive strategies.

Cancer patients often suffer from stress and psychosocial problems [4]. Often medical professionals focus primarily on the physical aspects and side effects of treatment rather than on the impact on mental health that results from stress and depression [5]. Given the varied sources of stress that negatively affect the treatment, recovery and health-related quality of life (HRQoL), methods to increase overall QoL should be considered as a part of treatment for breast cancer survivors [6].

Interestingly, in some studies it has been suggested that spousal or social supports buffer the adverse effect of stress on the HRQoL of breast cancer survivors. The absence of spousal support limited the positive effects of a healthy lifestyle and the presence of a spouse was found to be a main source of social support by 97.6% of breast cancer patients [6,7]. Additionally, several other studies have reported a buffering effect of spousal or social support against stress in breast cancer survivors [8,9,10,11].

A number of reports have investigated the factors associated with the improvement of HRQoL of breast cancer patients. Some studies reported that maintaining a healthy dietary habit after breast cancer diagnosis improved physical functions and HRQoL and lowered rates of cancer recurrence and mortality [12,13]. Interestingly, in a recent study the adherence to the American Cancer Society (ACS) nutrition and physical activity guideline for cancer survivors was found to be associated with a better HRQoL [13]. The ACS gathered a group of experts in nutrition, physical activity, and cancer survivorship to evaluate the scientific evidence and best clinical practices related to optimal nutrition and physical activity for cancer survivors. Patients who have survived cancer are often more likely to improve treatment outcomes by making changes to food choices, exercise, and dietary supplements in hopes of increasing both Qol and overall survival during recovery [14].

In addition to lifestyle choices, breastfeeding has been discussed as a preventive factor that decreases breast cancer incidence [15,16]. This implies that breastfeeding plays some role in the pathogenesis of breast cancer. There are reports that the breastfeeding experience improves the QoL in healthy women [3]; however, the effect of breastfeeding on the QoL of breast cancer survivors specifically has not been evaluated. Furthermore, the lower HRQoL in Korean breast cancer patients compared to other countries warrants the investigation of the specific factors modulating stress associated with HRQoL in Korean breast cancer patients specifically [17]. To investigate questions of HRQol in this population, we conducted a cross-sectional study to evaluate the potential mediating or moderating roles of spousal support, ACS guideline adherence, and breastfeeding experience between stress and physical and mental HRQoL in Korean breast cancer survivors.

## 2. Materials and Methods

### 2.1. Participants

One hundred and sixty three women who were breast cancer survivors were enrolled in the study between June 2016 and May 2018 at the Konkuk University hospital in Seoul, Korea. ‘Cancer survivor’ was defined identically to a previous study [18]. Participants were recruited based on the following inclusion criteria: (1) over one year had passed since the date of breast cancer surgery, and (2) the primary infiltrative breast cancer was without remote metastasis according to the American Joint Committee on Cancer classification of staging.

Of the 163 participants enrolled, those who had passed less than one year since their surgery (*n* = 5), those whose breast cancer was not primary (*n* = 6), those whose medical data was not available (*n* = 2), and those whose cancer had spread or recurred before enrollment (*n* = 6) were excluded from the study. As a result, 144 breast cancer survivors were included for analysis.

The sample size was calculated as 10 to 15 per observed variable, meeting the criteria of sufficient sample size according to the maximum-likelihood classification. This research was approved by the Institutional Review Board of the Konkuk University hospital (KUH1020068) and written informed consent was obtained from all participants.

### 2.2. Data Collection

The general characteristics of breast cancer survivors including breastfeeding experience, stress levels, HRQoL, and spousal support were collected through structured questionnaires. A three-day dietary diary was used to assess the participants’ diet for estimation of ACS guideline adherence. Information about breast cancer diagnosis, date of surgery, and stage of breast cancer were obtained by review of medical records.

### 2.3. Structured Questionnaires

To determine stress level, a self-assessment tool created by Danial and Georges and translated into the Korean language was used [19]. The assessment is composed of 3 categories: (1) psychological causes of stress, (2) bioecological causes of stress, and (3) personality factors that cause stress (score range: 5–25). The reliability estimations of the tool were calculated as Cronbach’s Alpha 0.81 and were similar to a previous Korean report which reported a value of 0.82 [20]. HRQoL was measured using the 36-item Short Form Health Survey (SF-36), which uses a domain-based score calculation method. This measure was used in a previous study which found that higher scores implied higher HRQoL [21]. Cronbach’s Alpha in this study was 0.81, and was reported as 0.93 in a previous Korean study [22]. Adherence to the ACS guideline (2012) was estimated by the following categories: (1) achieving and maintaining a healthy body weight using body mass index, (2) engaging in regular physical activity (MET-hours/week), and (3) following the ACS guidelines for cancer prevention (including achievement of a dietary pattern that is high in vegetables, fruits, whole grains, low in processed and red meat, low alcohol intake, and non-smoking). The detailed scoring was described in a previous study by our group [13]. The score range of ACS guideline adherence was defined between 3 and 12.

A spousal support assessment tool used in a previous study [23] was modified and applied to this study. It was composed of a total of 13 questions measuring love, respect, and trust towards one’s spouse. Higher scores meant higher spousal support (score range: 13–65). In a previous study [23], Cronbach’s Alpha was reported as 0.96 and was calculated as 0.94 the current study.

### 2.4. Data Analysis

Data was analyzed using SPSS version 20.0 and AMOS version 18.0, with statistical significance set at *p* < 0.05. The general characteristics, stress level, level of HRQoL, ACS guideline adherence, spousal support, and breastfeeding duration were analyzed using descriptive statistics. High and low ACS guidelines adherence scores were defined by median score values. The relationships between stress level, spousal support, ACS guideline adherence, and the two core domains of HRQoL (physical and mental) were analyzed using Pearson’s correlation coefficient.

The relationships between stress level, the level of HRQoL, ACS guideline adherence, and spousal support were analyzed using structural equation modeling. The variables used in structural equations were selected based on their significant relationships in the individual simple analysis for each effect channel, where it was required for them to support the theoretical model as well as form an adequate model in terms of statistical goodness-of-fit. The path coefficient was reported using the formula of dividing the estimates with the error after bootstrapping. Goodness of fit was calculated as 0.450.

In the structural relationship between stress and HRQoL level, the mediating effect channel through spousal support was examined. In addition, the moderating effect channel through ACS guideline adherence (high/low, based on median score) and breastfeeding experience (yes/no) was tested using multiple group analysis. The restricted model and the non-restricted model were established, and the models were interpreted as significantly “different” when the differences in the χ^2^ value were above the threshold. A *p*-value of less than 0.05 were considered statistically significant.

## 3. Results

### 3.1. Demographic Characteristics, Stress Level, the Level of HEQoL, Spousal Support, and ACS Guideline Adherence

The mean age of all 144 participants was 48.2 ± 8.8 years at the point of breast cancer diagnosis, and current mean age at enrollment was 51.0 ± 8.5 years. The most frequent period since breast cancer diagnosis was 1 to 5 years (57.0%, *n* = 82), and those above 5 years constituted 43.0% (*n* = 62). Of all participants, 91.3% had experience of giving birth, while 73.6% had experience breastfeeding with 16.12 weeks of duration. Patients were found to have breast cancer stage I (*n* = 73, 50.7%), stage II (*n* = 62, 43.1%), and stage III (*n* = 9, 6.2%). More detailed general characteristics are reported in Table 1.

The mean scores of stress level, ACS guideline adherence, and spousal support were 20.9 ± 3.41 (range: 5–25), 7.91 ± 11.92 (range: 3–12), and 33.41 ± 13.26 (range 13–65), respectively. Mean scores of the 8 subcategories of HRQoL varied between the range of 58.12–81.88 (scoring: zero to 100). The subcategory scores are shown in Table 2. As the median score of ACS guideline adherence was 8, the patients were dichotomized into high (*n* = 66, 45.8%) and low (*n* = 78, 54.2%) ACS adherence groups.

### 3.2. Correlation Analysis of Stress Level, Spousal Support, ACS Guideline Adherence, and HRQoL Scores

In the correlation analysis, stress was found to have a significant relationship with most of subcategories of HRQoL. Interestingly, all of the physical and mental QoL subcategories were significantly correlated with each other. ACS guideline adherence and spousal support were not directly correlated with any of HRQoL subcategories (Table 3).

### 3.3. Structural Equation Model Analysis Between Stress Level, HRQoL, Spousal Support, ACS Guideline Adherence, and Breastfeeding

The structural equation model analysis was performed to determine any mediating or moderating roles of HRQoL, spousal support, ACS guideline adherence, and breastfeeding experience in the relation between the levels of stress and HRQoL.

#### 3.3.1. Path Coefficient Analysis of Stress Level, HRQoL, and Spousal Support

Table 4 indicates that stress has negative effects on spousal support (β = −0.22, *p* = 0.015), physical quality of life (β = −0.60, *p* < 0.001), and mental quality of life (β = −0.76, *p* < 0.001). Additionally, spousal support positively affected physical quality of life (β = 0.27, *p* = 0.004) and mental quality of life (β = 0.40, *p* < 0.001). These findings indicate that the higher stress levels reduce physical and mental QoL, while higher spousal support leads to higher physical and mental QoL.

#### 3.3.2. Mediating Effect of Spousal Support in the Structural Relationship between Stress and HRQoL

**Figure 1:**
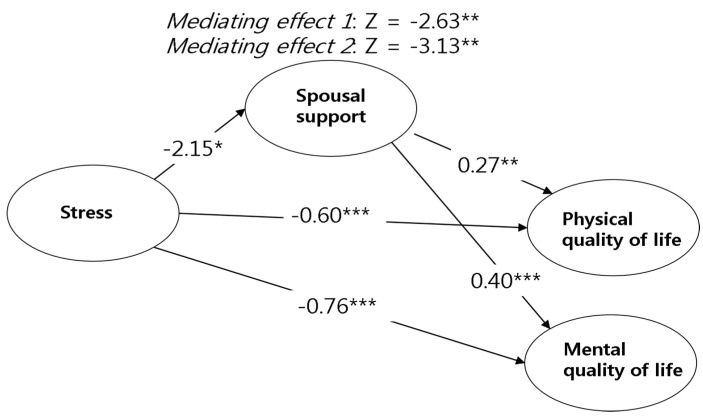
Mediating effects of spousal support in the relationship between stress and health-related quality of life (β); * *p* < 0.050; ** *p* < 0.010; *** *p* < 0.001.

In the relationship between the stress and the two main subsectors of HRQoL, the differences in the impact passage depending on the mediating effect of the spousal support were analyzed using the Sobel-test. As a result, the significant mediating effect of spousal support between the stress and physical QoL (Z = −2.63, *p* = 0.008) and the mental QoL (Z = −3.13, *p* < 0.002) were found. Results indicate that a mediating effect of spousal support is involved in the relationship between higher stress levels and lower physical and mental QoL. These relationships are shown in Figure 1.

When testing the association between the cancer survivors’ stress level, HRQoL, the ACS guideline adherence, and spousal support, the goodness-of-fit of the research model showed χ^2^ = 0.45, df = 18, *p* < 0.001, comparative fit index (CFI) = 0.92, incremental fit index (IFI) = 0.92, normal fit index (NFI) = 0.84, Tucker Lewis index (TLI) = 0.91, and root mean square error of approximation (RMSEA) = 0.08, indicating an acceptable goodness-of-fit level. 

#### 3.3.3. Moderating Effects of ACS Guideline Adherence in the Structural Relationship between Stress, Spousal Support, and HRQoL

The differences in the impact passage depending on the moderating effects of ACS guideline adherence (high/low) were tested using multiple-group analysis. Table 5 reports the moderating effects of ACS guideline adherence in the impact passages using the bootstrapping test. A significant difference between the groups of ACS guidelines adherence (*t* = −2.37, *p* = 0.014, high: β = −0.10; low: β = −0.31) was found in the impact of stress on spousal support (stress → spousal support). A similar difference was found in the relationship between the stress and the physical QoL (*t* = −3.27, *p* = 0.007, high adherence: β = −0.23; low adherence: β = −0.68) and the mental QoL (*t* = −3.86, *p* = 0.004, high adherence: β = −0.14; low adherence: β = −0.79). In other words, results indicate that spousal support, physical, and mental QoL decrease as stress increases, and these associations are stronger in the lower ACS guideline adherence group compared to those with high ACS guideline adherence. In the association between spousal support and physical QoL, a difference based on the group of ACS guideline adherence was found (*t* = 3.28, *p* = 0.009, high: β = −0.46; low: β = 0.18). However, there was no significant difference in the relationship between spousal support and mental QoL.

**Figure 2:**
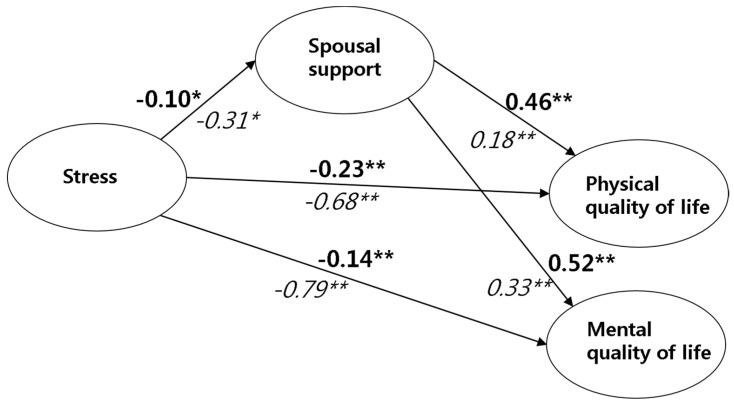
Moderating effects of American Cancer Society nutrition and physical activity guidelines for cancer survivors (ACS guideline) adherence on spousal support in the relationship between stress and health-related quality of life (β); * *p* < 0.050; ** *p* < 0.010; **Bold**: standardized estimated β coefficient of the high ACS guideline adherence group; *Italic*: standardized estimated β coefficient of the low ACS guideline adherence group.

Taken together, these results imply that HRQoL is increased in the lower stress and higher spousal support groups, while HRQoL would be improved much higher when the ACS adherence is higher simultaneously. The result is summarized in Figure 2.

#### 3.3.4. Moderating Effects of Breastfeeding Experience in the Structural Relationship between Stress, Spousal Support, and HRQoL

**Figure 3:**
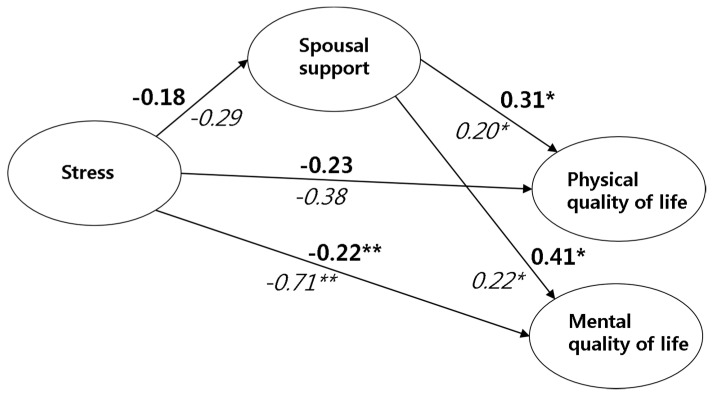
Moderating effects of breastfeeding experience on spousal support in the relationship between stress and health-related quality of life (β); * *p* < 0.050; ** *p* < 0.010; **Bold**: standardized estimated β coefficient of the breastfeeding experience (+) group; *Italic*: standardized estimated β coefficient of the breastfeeding experience (-) group.

Significant moderating effects of breastfeeding experience (yes/no) were found in the relationship between stress and estimates of spousal support, and the physical and mental QoL (Table 6). Breastfeeding experience did not modify the associations between stress, spousal support, and physical QoL (*p* > 0.050). This suggests that those with higher stress levels had lower spousal support and physical QoL, regardless of breastfeeding experience. However, in the relationship between the stress and mental QoL, breastfeeding experience was found to have a moderating effect (*t* = −3.27, *p* = 0.001, yes: β = −0.22; no: β = −0.71), indicating that the negative effect of stress on the mental QoL was stronger in women without breastfeeding experience. Moreover, women with breastfeeding experience had stronger positive effects of spousal support on the physical (*t* = 2.02, *p* = 0.041, yes: β = 0.31; no: β = −0.20) and mental QoL (*t* = 2.13, *p* = 0.038, yes: β = 0.41; no: β = 0.22). These relationships are demonstrated in Figure 3.

## 4. Discussion

In the present study, stress was found as a strong factor in decreasing the HRQoL of breast cancer survivors. However, finding and directly modulating the causes of stress would prove challenging. Thus, investigating alternative ways to reduce the adverse effects of stress on the HRQoL would improve the prognosis and HRQoL of breast cancer survivors.

In the current study, several factors modulating the adverse effects of stress were tested. First, spousal support was found as a significant mediating factor to reduce the adverse effects of stress on HRQoL despite the finding that it was not as strong an effect as the stress itself. It suggested that the previously found buffering effect of spousal support might be through the mediating effect. In a systemic review, high social support was found to lower stress levels and improve HRQoL in long-term breast cancer survivors [24]. The authors suggested that the social support for cancer patients from family and friends helped the successful treatments of cancer and increased positive motivation for healthy lifestyles [24]. Furthermore, daily spousal support was reported to be an important contributor to the daily emotional and physical wellbeing of women with both of early and advanced stage breast cancers [9,10]. However, the buffering effect of the spousal support was attenuated when breast cancer-related emotional and physical concerns reached high levels [9]. Therefore, a specific plan to encourage spousal support through counseling or educational programs for patients and their spouses would be warranted.

ACS guideline adherence was found to have a moderating effect on both the adverse effect of stress on HRQoL and on the buffering effects of spousal support. Numbers of studies reported that adherence to the ACS guideline in the breast cancer patients reduced cancer recurrence and mortality [12,13,25]. Interestingly, an association between ACS guideline adherence and the QoL of cancer patients has been reported as well; the higher healthy daily habits scores were related to the higher HRQoL levels [7,12,25]. Furthermore, a Korean study investigated the relationship between uncertainty and QoL in younger breast cancer patients and found a mediating effect of spousal intimacy on feelings of uncertainty [26]. As uncertainty may be negatively associated with the QoL of breast cancer patients, there would be a possibility of a mediating effect of spousal support in the relationship between the stress and HRQoL as shown in the current study [27]. However, there are only limited evidences that tested the potential interrelations between various elements such as stress, ACS guidelines adherence, HRQoL, and spousal support.

In the present study, we report a moderating effect of breastfeeding; the group with no breastfeeding experience had a significant decrease in mental QoL in breast cancer survivors. Though previous studies have indicated that longer breastfeeding periods could lower the incidence or mortality rates of breast cancer, there have been no prior studies to date on the effect of breastfeeding on the relationship between stress, spousal support, and mental QoL in breast cancer survivors [15,28]. Breastfeeding is well-known to play important roles in maternal recovery from pregnancy and to determine multiple aspects of maternal health in later life [29]. Moreover, the immediate psychological effect of breastfeeding has been shown to reduce emotional stress and anxiety by the action of various endogenous hormonal system [30,31]. However, there have been no study of the long-term effects of breastfeeding on stress.

Interestingly, there are few studies that investigate the breastfeeding experience and QoL in healthy women. Chen et al. reported a positive association between HRQoL and breastfeeding duration; mothers who continued breastfeeding for longer than six months obtained significantly higher QoL scores [32]. Similarly, other research groups reported better QoL in mothers who have breastfed [3,33]. Moreover, the authors suggested that breastfeeding would affect the QoL as successful breastfeeding depends on both physiological and psychological factors which, along with social relationships, are important components of QoL [3]. Hence, further research on the relationship between the mental QoL and breastfeeding in breast cancer survivors were warranted. The findings of the current study highlight the importance of developing an active educational program to increase the awareness of the importance of breastfeeding in the women of childbearing age [34].

To our knowledge, this is the first study suggesting that the formerly reported buffering effect of spousal support would be enhanced by ACS guideline adherence and that the breastfeeding experience and ACS guideline adherence would be able to improve HRQoL in breast cancer survivors. However, there are several limitations in the current study. First, this was a cross-sectional study and the relationships between HRQoL and other factors were not necessarily cause and effect relationships. Moreover, there could have been recall bias as large portions of data were collected by self-report questionnaires. Thus, long-term prospective study is warranted to find the key factors that could improve the QoL of cancer survivors. The number of enrolled subjects was not large enough to claim the generalizable conclusion for the effects of the investigated factors as this was a single center study. Moreover, the patients showed relatively high scores of ACS adherence (mean score = 7.91 of 13). Because the score was highly distributed, the references to World Health Organization (WHO) recommendations on the physical activities could not be used as a cut-off score. It seems to be because of the limitation of single center studies. Thus, it would be necessary to investigate the factors enrolling more numbers of subjects as a multicenter study to represent general Korean breast cancer survivors in a further study. In addition, only spousal support, ACS guideline adherence, and breastfeeding were investigated as factors influencing the relationship between stress and HRQoL. However, there would be other various factors that could influence the effects of stress including treatment modalities and clinicopathologic characteristics such as the effects of different cancer stages. Thus, a prospective study enrolling more numbers of breast cancer patients with various target factors would be warranted in the future.

## 5. Conclusions

This study provides evidence of significant mediating roles of spousal support in the relationship between stress and HRQoL in breast cancer survivors; spousal support buffered the adverse effect of stress on HRQoL. Moreover, ACS guideline adherence and breastfeeding experience were found to moderate the adverse effect of stress on HRQoL and the buffering effect of spousal support. Therefore, to improve HRQoL of breast cancer survivors, specific plans should be outlined to encourage spousal support, adherence to ACS guidelines, and breastfeeding at a childbearing age. These findings support the need for development of counseling and educational programs to address these factors as well as the management of emotional stress.

## Figures and Tables

**Table 1:** General characteristics of participants (*n* = 144).

Characteristics	Categories	*n*	ValidPercentage (%)	CumulativePercentage (%)
Time since surgery	1–2 years	27	18.8	18.8
	2–3 years	19	13.2	32.0
	3–4 years	17	11.8	43.8
	4–5 years	19	13.2	57.0
	>5 years	62	43.0	100.0
Marital status	Married or cohabitation	109	76.5	76.5
	Not married or divorced or widowed	35	23.5	100.0
Breastfeeding experience	No	38	26.4	26.4
	Yes	106	73.6	100
Parity	No	13	8.7	8.7
	Yes	136	91.3	100.0
Education level	Elementary school or below	25	17.4	17.4
	Middle school	15	10.4	27.8
	High school	71	49.3	77.1
	College or above	33	22.9	100.0
Cancer stage	I	73	50.7	50.7
	II	62	43.1	93.8
	III	9	6.2	100.0

**Table 2:** Descriptive statistics of stress level, health-related quality of life, spousal support, and American Cancer Society (ACS) adherence (*n* = 144).

Variables		Mean ± SD	Skewness	Kurtosis	Min-Max
Stress level		20.9 ± 3.41	1.24	1.40	5–25
HRQoL					
Physical QoL	PF	73.45 ± 22.29	−0.94	0.32	5–100
	RP	75.21 ± 26.20	−1.03	0.35	0–100
	BP	69.75 ± 24.10	−0.24	−0.99	21–100
	GH	60.65 ± 19.99	−0.54	−0.12	5–100
Mental QoL	VT	58.12 ± 20.95	−0.35	−0.64	0–100
	SF	81.88 ± 21.28	−1.10	0.43	25–100
	RE	77.46 ± 26.36	−1.04	0.15	0–100
	MH	69.31 ± 20.43	−0.77	0.52	0–100
Spousal support		33.41 ± 13.26	0.45	−0.60	13–65
ACS adherence		7.91 ± 1.91	0.04	−0.29	3–12

ACS adherence = adherence to the American Cancer Society nutrition and physical activity guideline for cancer survivors; BP = Bodily pain; HRQoL = Health-related quality of life; MH = Mental health; PF = Physical functioning; QoL = Quality of life; RE = Role emotional; RP = Role physical; SD = Standard deviation; SF = Social functioning; VT = Vitality.

**Table 3:** Pearson’s correlation analysis of stress level, spousal support, ACS guideline adherence, and health-related quality of life.

		Stress	Spousal Support	ACS	Physical Quality of Life				Mental Quality of Life			
					PF	RP	BP	GH	PF	RP	BP	GH
	Stress	1.000										
	Spousal support	0.151	1.000									
	ACS	0.031	0.057	1.000								
Pysical QoL	PF	−0.415 *	0.049	0.032	1.000							
	RP	−0.494 *	0.040	0.121	0.594 *	1.000						
	BP	−0.506 *	−0.044	−0.051	0.504 *	0.585 *	1.000					
	GH	−0.460 *	−0.140	0.087	0.393 *	0.405 *	0.448 *	1.000				
Mental QoL	VT	−0.536 *	−0.069	−0.059	0.443 *	0.433 *	0.559 *	0.643 *	1.000			
	SF	−0.593 *	−0.041	0.051	0.528 *	0.684 *	0.592 *	0.473 *	0.549 *	1.000		
	RE	−0.609 *	−0.026	0.075	0.607 *	0.821 *	0.556 *	0.417 *	0.487 *	0.685 *	1.000	
	MH	−0.679 *	−0.151	0.110	0.369 *	0.491 *	0.495 *	0.524 *	0.654 *	0.580 *	0.570 *	1.000

* *p* < 0.050; ACS = Adherence to the American Cancer Society nutrition and physical activity guideline for cancer survivors; BP = Bodily pain; HRQoL = Health-related quality of life; MH = Mental health; PF = Physical functioning; RE = Role emotional; RP = Role physical; SF = Social functioning; VT = Vitality.

**Table 4:** Path coefficient analysis results of internal model.

			β	S.E	Min	Max	*t*	*p*
Stress level	→	spousal support	−0.22	0.06	−0.04	−0.40	−3.77	0.015 *
Stress level	→	physical quality of life	−0.60	0.06	−0.72	−0.51	−10.68	<0.001 ***
Stress level	→	mental quality of life	−0.76	0.04	−0.83	−0.68	−20.15	<0.001 ***
Spousal support	→	physical quality of life	0.27	0.11	0.11	0.29	2.33	0.004 **
Spousal support	→	mental quality of life	0.40	0.07	−0.14	0.15	5.61	<0.001 ***

β: standardized estimated coefficient’ * *p* < 0.050; ** *p* < 0.010; *** *p* < 0.001.

**Table 5:** Multiple group analysis results by ACS adherence (moderating effect).

			ACS Adherence (β)		*t*	df	*p*
			High	Low			
Stress	→	spousal support	−0.10	−0.31	−2.37	105	0.014 *
Stress	→	physical quality of life	−0.23	−0.68	−3.27	105	0.007 **
Stress	→	mental quality of life	−0.14	−0.79	−3.86	105	0.004 **
Spousal support	→	physical quality of life	0.46	0.18	3.28	105	0.009 **
Spousal support	→	mental quality of life	0.52	0.33	1.79	105	0.062

ACS adherence = Adherence to the American Cancer Society nutrition and physical activity guideline for cancer survivors; β: standardized estimated coefficient; * *p* < 0.050; ** *p* < 0.010.

**Table 6:** Multiple group analysis results by breastfeeding experience (moderating effect).

			Breastfeeding (β)		*t*	df	*p*
			Yes	No			
Stress	→	spousal support	−0.18	−0.29	−1.05	105	0.153
Stress	→	physical quality of life	−0.23	−0.38	−1.41	105	0.104
Stress	→	mental quality of life	−0.22	−0.71	−3.27	105	0.001 **
Spousal support	→	physical quality of life	0.31	0.20	2.02	105	0.041 *
Spousal support	→	mental quality of life	0.41	0.22	2.13	105	0.038 *

β: standardized estimated coefficient; * *p* < 0.050; ** *p* < 0.010.
